# Supplementary material for: Rapid Epidemiological Analysis of Comorbidities and Treatments as risk factors for COVID-19 in Scotland (REACT-SCOT): A population-based case-control study
Source: PLoS Med. 2020 Oct 20;17(10):e1003374. doi: 10.1371/journal.pmed.1003374 (PMC7575101; doi:10.1371/journal.pmed.1003374)
Supplement: S1 Table — (PDF) [file pmed.1003374.s001.pdf]

Table S1. Associations of severe disease with listed conditions in those aged less than 60

			Univariate		Multivariable	
	Controls (4738)	Cases (475)	Rate ratio (95% CI)	p-value	Rate ratio (95% CI)	p-value
Care home	4 (0%)	20 (4%)	65.0 (19.3, 218.8)	2×10^{-11}	25.5 (6.9, 94.9)	1×10^{-6}
Any prescription	3227 (68%)	418 (88%)	3.74 (2.79, 5.01)	9×10^{-19}	2.12 (1.55, 2.90)	3×10^{-6}
Any admission	1617 (34%)	275 (58%)	2.78 (2.28, 3.39)	3×10^{-24}	1.49 (1.18, 1.88)	7×10^{-4}
Type 1 diabetes	46 (1%)	14 (3%)	3.70 (2.01, 6.79)	3×10^{-5}	1.84 (0.92, 3.69)	0.08
Type 2 diabetes	253 (5%)	77 (16%)	3.70 (2.80, 4.90)	6×10^{-20}	2.59 (1.90, 3.53)	2×10^{-9}
Other/unknown type	26 (1%)	18 (4%)	8.1 (4.4, 14.9)	2×10^{-11}	6.9 (3.5, 13.7)	4×10^{-8}
Ischaemic heart disease	128 (3%)	34 (7%)	2.88 (1.93, 4.29)	2×10^{-7}	1.25 (0.79, 1.99)	0.3
Other heart disease	182 (4%)	73 (15%)	4.67 (3.47, 6.29)	3×10^{-24}	1.74 (1.20, 2.52)	0.004
Asthma or chronic airway disease	616 (13%)	136 (29%)	2.73 (2.19, 3.40)	3×10^{-19}	1.74 (1.36, 2.22)	1×10^{-5}
Chronic kidney disease or transplant recipient	9 (0%)	16 (3%)	19.4 (8.3, 45.5)	8×10^{-12}	5.9 (2.0, 16.9)	0.001
Neurological (except epilepsy) or dementia	64 (1%)	50 (11%)	8.7 (5.9, 12.8)	1×10^{-27}	3.16 (1.99, 5.03)	1×10^{-6}
Liver disease	21 (0%)	10 (2%)	4.76 (2.24, 10.11)	5×10^{-5}	1.69 (0.72, 3.98)	0.2
Immune deficiency or suppression	20 (0%)	14 (3%)	7.2 (3.6, 14.4)	2×10^{-8}	1.59 (0.59, 4.28)	0.4
