# Supplementary material for: Rapid Epidemiological Analysis of Comorbidities and Treatments as risk factors for COVID-19 in Scotland (REACT-SCOT): A population-based case-control study
Source: PLoS Med. 2020 Oct 20;17(10):e1003374. doi: 10.1371/journal.pmed.1003374 (PMC7575101; doi:10.1371/journal.pmed.1003374)
Supplement: S2 Table — (PDF) [file pmed.1003374.s002.pdf]

Table S2. Associations of severe disease with listed conditions in those aged 60-74 years

	Controls (8734)	Cases (881)	Univariate		Multivariable	
			Rate ratio (95% CI)	*p*-value	Rate ratio (95% CI)	*p*-value
Care home	90 (1%)	176 (20%)	40.8 (28.6, 58.4)	3×10^{-92}	25.3 (17.2, 37.2)	7×10^{-61}
Any prescription	7591 (87%)	839 (95%)	3.23 (2.33, 4.48)	2×10^{-12}	1.53 (1.08, 2.17)	0.02
Any admission	4394 (50%)	674 (77%)	3.33 (2.82, 3.92)	8×10^{-47}	1.99 (1.64, 2.41)	3×10^{-12}
Type 1 diabetes	42 (0%)	8 (1%)	2.13 (0.99, 4.57)	0.05	0.97 (0.38, 2.51)	1
Type 2 diabetes	1319 (15%)	219 (25%)	1.90 (1.61, 2.25)	3×10^{-14}	1.57 (1.30, 1.90)	3×10^{-6}
Other/unknown type	73 (1%)	8 (1%)	1.20 (0.58, 2.51)	0.6	1.28 (0.57, 2.87)	0.5
Ischaemic heart disease	955 (11%)	170 (19%)	1.96 (1.63, 2.35)	5×10^{-13}	1.15 (0.92, 1.43)	0.2
Other heart disease	1236 (14%)	265 (30%)	2.66 (2.27, 3.12)	2×10^{-33}	1.19 (0.97, 1.46)	0.09
Asthma or chronic airway disease	1686 (19%)	328 (37%)	2.51 (2.17, 2.91)	6×10^{-34}	1.82 (1.53, 2.16)	7×10^{-12}
Chronic kidney disease or transplant recipient	30 (0%)	24 (3%)	8.0 (4.7, 13.8)	5×10^{-14}	4.90 (2.59, 9.25)	1×10^{-6}
Neurological (except epilepsy) or dementia	321 (4%)	177 (20%)	6.7 (5.5, 8.2)	1×10^{-74}	2.36 (1.82, 3.06)	1×10^{-10}
Liver disease	53 (1%)	21 (2%)	4.04 (2.42, 6.74)	9×10^{-8}	2.22 (1.24, 3.97)	0.007
Immune deficiency or suppression	47 (1%)	15 (2%)	3.19 (1.78, 5.70)	9×10^{-5}	1.43 (0.71, 2.89)	0.3
