# Supplementary material for: Rapid Epidemiological Analysis of Comorbidities and Treatments as risk factors for COVID-19 in Scotland (REACT-SCOT): A population-based case-control study
Source: PLoS Med. 2020 Oct 20;17(10):e1003374. doi: 10.1371/journal.pmed.1003374 (PMC7575101; doi:10.1371/journal.pmed.1003374)
Supplement: S3 Table — (PDF) [file pmed.1003374.s003.pdf]

Table S3. Associations of severe disease with listed conditions in those aged 75 years and over

	Controls (23476)	Cases (2916)	Univariate		Multivariable	
			Rate ratio (95% CI)	p-value	Rate ratio (95% CI)	p-value
Care home	2841 (12%)	1698 (58%)	19.3 (17.2, 21.7)	2×10^{-539}	13.9 (12.3, 15.7)	6×10^{-372}
Any prescription	22665 (97%)	2872 (98%)	2.30 (1.69, 3.14)	1×10^{-7}	1.13 (0.80, 1.60)	0.5
Any admission	16527 (70%)	2508 (86%)	2.50 (2.24, 2.79)	5×10^{-59}	1.34 (1.17, 1.53)	2×10^{-5}
Type 1 diabetes	70 (0%)	21 (1%)	2.73 (1.67, 4.49)	7×10^{-5}	1.63 (0.90, 2.97)	0.1
Type 2 diabetes	3970 (17%)	613 (21%)	1.38 (1.25, 1.53)	1×10^{-10}	1.26 (1.12, 1.41)	8×10^{-5}
Other/unknown type	184 (1%)	22 (1%)	1.16 (0.74, 1.81)	0.5	0.95 (0.56, 1.61)	0.8
Ischaemic heart disease	4392 (19%)	702 (24%)	1.35 (1.23, 1.48)	4×10^{-10}	1.07 (0.96, 1.20)	0.2
Other heart disease	7192 (31%)	1411 (48%)	2.03 (1.87, 2.20)	2×10^{-66}	1.37 (1.24, 1.53)	2×10^{-9}
Asthma or chronic airway disease	5306 (23%)	970 (33%)	1.73 (1.59, 1.88)	6×10^{-36}	1.43 (1.29, 1.58)	6×10^{-12}
Chronic kidney disease or transplant recipient	163 (1%)	57 (2%)	2.67 (1.94, 3.67)	2×10^{-9}	2.19 (1.49, 3.20)	6×10^{-5}
Neurological (except epilepsy) or dementia	2897 (12%)	1154 (40%)	5.00 (4.56, 5.48)	2×10^{-263}	1.88 (1.68, 2.10)	3×10^{-29}
Liver disease	59 (0%)	20 (1%)	2.88 (1.72, 4.83)	6×10^{-5}	1.45 (0.77, 2.76)	0.3
Immune deficiency or suppression	76 (0%)	11 (0%)	1.26 (0.66, 2.40)	0.5	1.24 (0.59, 2.58)	0.6
