# Supplementary material for: Rapid Epidemiological Analysis of Comorbidities and Treatments as risk factors for COVID-19 in Scotland (REACT-SCOT): A population-based case-control study
Source: PLoS Med. 2020 Oct 20;17(10):e1003374. doi: 10.1371/journal.pmed.1003374 (PMC7575101; doi:10.1371/journal.pmed.1003374)
Supplement: S4 Table — (PDF) [file pmed.1003374.s004.pdf]

Table S4. Comparison of severe non-fatal and fatal cases, by test positive status and entry to critical care

	Non-fatal	Fatal		
		Test-positive		No positive test
	Critical care, non-fatal (483)	Critical care, fatal (250)	No critical care, fatal (2213)	No critical care, fatal (1307)
Age [median (IQR)]	59 (51-65)	65 (58-73)	83 (77-88)	84 (76.5-89)
Males	323 (67%)	194 (78%)	1104 (50%)	585 (45%)
Care home	4 (1%)	1 (0%)	1051 (47%)	838 (64%)
Any prescription	431 (89%)	232 (93%)	2171 (98%)	1278 (98%)
Any admission	264 (55%)	165 (66%)	1928 (87%)	1091 (83%)
Type 1 diabetes	10 (2%)	2 (1%)	22 (1%)	8 (1%)
Type 2 diabetes	93 (19%)	63 (25%)	501 (23%)	251 (19%)
Other/unknown type	13 (3%)	7 (3%)	21 (1%)	7 (1%)
Ischaemic heart disease	47 (10%)	33 (13%)	557 (25%)	269 (21%)
Other heart disease	67 (14%)	46 (18%)	1061 (48%)	570 (44%)
Asthma or chronic airway disease	121 (25%)	66 (26%)	798 (36%)	444 (34%)
Chronic kidney disease or transplant recipient	7 (1%)	6 (2%)	61 (3%)	23 (2%)
Neurological (except epilepsy) or dementia	20 (4%)	18 (7%)	801 (36%)	542 (41%)
Liver disease	1 (0%)	4 (2%)	30 (1%)	15 (1%)
Immune deficiency or suppression	6 (1%)	4 (2%)	24 (1%)	6 (0%)
