# Supplementary material for: Rapid Epidemiological Analysis of Comorbidities and Treatments as risk factors for COVID-19 in Scotland (REACT-SCOT): A population-based case-control study
Source: PLoS Med. 2020 Oct 20;17(10):e1003374. doi: 10.1371/journal.pmed.1003374 (PMC7575101; doi:10.1371/journal.pmed.1003374)
Supplement: S5 Table — (PDF) [file pmed.1003374.s005.pdf]

Table S5. Associations of severe disease with hospital diagnoses by ICD chapter in last 5 years, in those without any listed condition

			Univariate		Multivariable	
	Controls (17793)	Cases (945)	Rate ratio (95% CI)	*p*-value	Rate ratio (95% CI)	*p*-value
Ch.I infectious	549 (3%)	78 (8%)	3.18 (2.31, 4.36)	1×10^{-12}	1.55 (1.07, 2.25)	0.02
Ch.II Neoplasms	1250 (7%)	97 (10%)	1.88 (1.45, 2.45)	3×10^{-6}	1.52 (1.14, 2.04)	0.004
Ch.III blood	361 (2%)	39 (4%)	2.22 (1.46, 3.40)	2×10^{-4}	1.15 (0.71, 1.84)	0.6
Ch.IV Endocrine	363 (2%)	56 (6%)	3.11 (2.11, 4.57)	8×10^{-9}	1.41 (0.91, 2.19)	0.1
Ch.V Mental	536 (3%)	120 (13%)	4.46 (3.34, 5.95)	5×10^{-24}	2.52 (1.80, 3.52)	7×10^{-8}
Ch.VI nervous	285 (2%)	21 (2%)	1.87 (1.12, 3.14)	0.02	1.16 (0.65, 2.07)	0.6
Ch.VII eye	1812 (10%)	89 (9%)	0.87 (0.67, 1.14)	0.3	0.78 (0.59, 1.04)	0.09
Ch.VIII ear	86 (0%)	5 (1%)	1.11 (0.40, 3.06)	0.8	0.74 (0.24, 2.28)	0.6
Ch.IX circulatory	541 (3%)	56 (6%)	1.78 (1.25, 2.55)	0.002	0.98 (0.66, 1.46)	0.9
Ch.X respiratory	432 (2%)	81 (9%)	4.10 (2.93, 5.74)	2×10^{-16}	2.32 (1.60, 3.36)	9×10^{-6}
Ch.XI digestive	2244 (13%)	158 (17%)	1.52 (1.24, 1.87)	7×10^{-5}	1.03 (0.81, 1.30)	0.8
Ch.XII skin	383 (2%)	40 (4%)	1.94 (1.29, 2.91)	0.001	1.24 (0.79, 1.94)	0.3
Ch.XIII musculoskeletal	1412 (8%)	118 (12%)	1.75 (1.37, 2.22)	6×10^{-6}	1.18 (0.90, 1.55)	0.2
Ch.XIV genitourinary	1355 (8%)	148 (16%)	2.59 (2.06, 3.26)	6×10^{-16}	1.41 (1.06, 1.87)	0.02
Ch.XV Pregnancy	22 (0%)	0 (0%)	0.00 (0.00, Inf)	1	0.00 (0.00, Inf)	1
Ch.XVII Congenital	29 (0%)	4 (0%)	1.91 (0.59, 6.23)	0.3	1.41 (0.40, 5.06)	0.6
Ch.XVIII Symptoms	2049 (12%)	209 (22%)	2.33 (1.91, 2.84)	9×10^{-17}	1.25 (0.97, 1.62)	0.08
Ch.XIX Injury	1193 (7%)	135 (14%)	2.05 (1.61, 2.62)	9×10^{-9}	1.15 (0.59, 2.23)	0.7
Ch.XX External	1341 (8%)	156 (17%)	2.21 (1.75, 2.78)	2×10^{-11}	1.01 (0.53, 1.93)	1
Ch.XXI Health factors	2048 (12%)	166 (18%)	1.73 (1.40, 2.13)	3×10^{-7}	0.99 (0.76, 1.28)	0.9
