# Supplementary material for: Rapid Epidemiological Analysis of Comorbidities and Treatments as risk factors for COVID-19 in Scotland (REACT-SCOT): A population-based case-control study
Source: PLoS Med. 2020 Oct 20;17(10):e1003374. doi: 10.1371/journal.pmed.1003374 (PMC7575101; doi:10.1371/journal.pmed.1003374)
Supplement: S6 Table — (PDF) [file pmed.1003374.s006.pdf]

Table S6. Associations of severe disease with prescribed drugs by BNF chapter in those without any listed condition

			Univariate		Multivariable	
	Controls (17793)	Cases (945)	Rate ratio (95% CI)	*p*-value	Rate ratio (95% CI)	*p*-value
BNF 1 Gastro	6611 (37%)	516 (55%)	2.14 (1.84, 2.50)	6×10^{-22}	1.46 (1.22, 1.75)	3×10^{-5}
BNF 2 Cardiovascular	9095 (51%)	507 (54%)	1.23 (1.04, 1.45)	0.01	1.01 (0.85, 1.20)	0.9
BNF 3 Respiratory	1318 (7%)	100 (11%)	1.55 (1.21, 1.99)	6×10^{-4}	1.09 (0.83, 1.43)	0.5
BNF 4 Nervous	7056 (40%)	584 (62%)	2.52 (2.15, 2.95)	4×10^{-30}	1.79 (1.49, 2.14)	4×10^{-10}
BNF 5 Infections	3780 (21%)	341 (36%)	2.17 (1.83, 2.56)	1×10^{-19}	1.63 (1.36, 1.95)	2×10^{-7}
BNF 6 Endocrine	2952 (17%)	195 (21%)	1.33 (1.09, 1.62)	0.005	0.99 (0.79, 1.23)	0.9
BNF 7 Obstetrics	2231 (13%)	106 (11%)	0.96 (0.75, 1.21)	0.7	0.67 (0.52, 0.87)	0.002
BNF 8 Malignant	356 (2%)	32 (3%)	2.19 (1.35, 3.56)	0.002	1.45 (0.87, 2.42)	0.2
BNF 9 Nutrition	3242 (18%)	309 (33%)	2.40 (1.99, 2.89)	2×10^{-20}	1.63 (1.33, 2.00)	3×10^{-6}
BNF 10 Musculoskeletal	3723 (21%)	250 (26%)	1.49 (1.25, 1.77)	5×10^{-6}	1.00 (0.83, 1.21)	1
BNF 11 Eye	2151 (12%)	117 (12%)	1.02 (0.80, 1.29)	0.9	0.80 (0.62, 1.04)	0.09
BNF 12 Ear	1693 (10%)	77 (8%)	1.01 (0.77, 1.31)	1	0.67 (0.50, 0.89)	0.006
BNF 13 Skin	3723 (21%)	292 (31%)	1.72 (1.45, 2.04)	3×10^{-10}	1.12 (0.93, 1.36)	0.2
BNF 14 Other	3884 (22%)	375 (40%)	2.43 (2.05, 2.88)	1×10^{-24}	1.81 (1.50, 2.20)	1×10^{-9}
