# Supplementary material for: Rapid Epidemiological Analysis of Comorbidities and Treatments as risk factors for COVID-19 in Scotland (REACT-SCOT): A population-based case-control study
Source: PLoS Med. 2020 Oct 20;17(10):e1003374. doi: 10.1371/journal.pmed.1003374 (PMC7575101; doi:10.1371/journal.pmed.1003374)
Supplement: S7 Table — (PDF) [file pmed.1003374.s007.pdf]

Table S7. Univariate associations of severe disease with hospital diagnoses by ICD subchapters in those without any listed conditions: rows retained are those with $p < 0.001$ and at least 50 cases and controls

	Controls (17793)	Cases (945)	Rate ratio (95% CI)	p-value
Ch I: A30-A49 Other Bacterial Diseases	106 (1%)	23 (2%)	6.3 (3.2, 12.4)	1×10^{-7}
Ch I: B95-B98 Bacterial And Viral Infectious Agents	231 (1%)	34 (4%)	2.71 (1.69, 4.33)	3×10^{-5}
Ch II: C15-C26 Malignant Neoplasms Of Digestive Organs	95 (1%)	15 (2%)	8.2 (3.7, 18.0)	2×10^{-7}
Ch II: C76-C80 Malignant Neoplasms Of Ill-Defined, Other Secondary And Unspecified Sites	110 (1%)	20 (2%)	5.4 (2.9, 10.1)	1×10^{-7}
Ch II: C81-C96 Malignant Neoplasms Of Lymphoid, Hematopoietic And Related Tissue	56 (0%)	13 (1%)	5.7 (2.5, 12.8)	3×10^{-5}
Ch II: D37-D48 Neoplasms Of Uncertain Behavior, Polycythemia Vera And Myelodysplastic Syndromes	73 (0%)	9 (1%)	5.3 (2.0, 14.4)	9×10^{-4}
Ch IV: E70-E88 Metabolic Disorders	225 (1%)	33 (3%)	2.72 (1.68, 4.41)	5×10^{-5}
Ch V: F01-F09 Mental Disorders Due To Known Physiological Conditions	227 (1%)	84 (9%)	10.5 (6.5, 17.0)	2×10^{-21}
Ch V: F30-F39 Mood [Affective] Disorders	58 (0%)	14 (1%)	4.94 (2.24, 10.92)	8×10^{-5}
Ch IX: I60-I69 Cerebrovascular Diseases	193 (1%)	27 (3%)	2.61 (1.49, 4.58)	8×10^{-4}
Ch X: J09-J18 Influenza And Pneumonia	200 (1%)	34 (4%)	3.47 (2.09, 5.75)	1×10^{-6}
Ch X: J20-J22 Other Acute Lower Respiratory Infections	163 (1%)	44 (5%)	7.2 (4.3, 12.0)	9×10^{-14}
Ch XII: L80-L99 Other Disorders Of The Skin And Subcutaneous Tissue	108 (1%)	17 (2%)	4.02 (2.05, 7.90)	5×10^{-5}
Ch XIV: N17-N19 Acute Kidney Failure And Chronic Kidney Disease	371 (2%)	58 (6%)	3.94 (2.63, 5.92)	4×10^{-11}
Ch XIV: N30-N39 Other Diseases Of The Urinary System	615 (3%)	87 (9%)	3.41 (2.48, 4.68)	5×10^{-14}
Ch XVIII: R25-R29 Symptoms And Signs Involving The Nervous And Musculoskeletal Systems	310 (2%)	67 (7%)	4.29 (2.87, 6.41)	1×10^{-12}
Ch XVIII: R40-R46 Symptoms And Signs Involving Cognition, Perception, Emotional State And Behavior	220 (1%)	47 (5%)	4.67 (2.99, 7.30)	1×10^{-11}
Ch XVIII: R50-R69 General Symptoms And Signs	492 (3%)	56 (6%)	2.02 (1.42, 2.89)	1×10^{-4}
Ch XVIII: R90-R94 Abnormal Findings On Diagnostic Imaging And In Function Studies, Without Diagnosis	168 (1%)	25 (3%)	2.87 (1.66, 4.96)	2×10^{-4}
Ch XIX: S70-S79 Injuries To The Hip And Thigh	248 (1%)	52 (6%)	3.18 (2.00, 5.05)	9×10^{-7}
Ch XX: W00-W19 Slipping, Tripping, Stumbling And Falls	763 (4%)	99 (10%)	2.42 (1.79, 3.27)	1×10^{-8}
Ch XXI: Z40-Z54 Encounters For Other Specific Health Care	730 (4%)	66 (7%)	1.88 (1.38, 2.58)	8×10^{-5}
Ch XXI: Z77-Z99 Persons With Potential Health Hazards Related To Family And Personal History And Certain Conditions Influencing Health Status	1373 (8%)	107 (11%)	1.62 (1.26, 2.07)	2×10^{-4}
