# Supplementary material for: Rapid Epidemiological Analysis of Comorbidities and Treatments as risk factors for COVID-19 in Scotland (REACT-SCOT): A population-based case-control study
Source: PLoS Med. 2020 Oct 20;17(10):e1003374. doi: 10.1371/journal.pmed.1003374 (PMC7575101; doi:10.1371/journal.pmed.1003374)
Supplement: S8 Table — (PDF) [file pmed.1003374.s008.pdf]

Table S8. Stepwise regression: variables retained in model for severe disease

	log rate ratio	*p*-value
Care/nursing home	2.29	8×10^{-160}
SIMD - quintile 1 as reference		
quintile 2	0.04	0.6
quintile 3	-0.08	0.3
quintile 4	-0.15	0.06
quintile 5 - least deprived	-0.29	0.002
Diabetes - non-diabetic as reference		
Type 1 diabetes	0.53	0.02
Type 2 diabetes	0.34	1×10^{-7}
Other/unknown type	0.43	0.2
Other heart disease	0.28	1×10^{-6}
Asthma or chronic airway disease	0.30	2×10^{-8}
Chronic kidney disease or transplant recipient	1.34	2×10^{-12}
Neurological (except epilepsy) or dementia	0.56	1×10^{-15}
Liver disease	0.61	0.008
Any admission	0.49	7×10^{-15}
Any prescription	0.26	0.03
BNF 1 Gastro	0.22	7×10^{-5}
BNF 4 Nervous	0.34	8×10^{-9}
BNF 5 Infections	0.16	0.002
BNF 6 Endocrine	0.15	0.006
BNF 9 Nutrition	0.30	2×10^{-8}
BNF 11 Eye	-0.19	0.005
BNF 14 Other	0.21	7×10^{-5}
