# Supplementary material for: Rapid Epidemiological Analysis of Comorbidities and Treatments as risk factors for COVID-19 in Scotland (REACT-SCOT): A population-based case-control study
Source: PLoS Med. 2020 Oct 20;17(10):e1003374. doi: 10.1371/journal.pmed.1003374 (PMC7575101; doi:10.1371/journal.pmed.1003374)
Supplement: Attachment — Submitted filename: response_reviewers.pdf [file pmed.1003374.s011.pdf]

PMEDICINE-D-20-02483R1: authors' response to reviewers' comments

Requests from the editors:

Please ensure that the paper adheres to the PLOS Data Availability Policy

We have included the following data availability statement on the submission portal and in the manuscript:

The component datasets used here are available via the Public Benefits Privacy Panel for Health at https://www.informationgovernance.scot.nhs.uk/pbpphsc/ for researchers who meet the criteria for access to confidential data. All source code used for derivation of variables, statistical analysis and generation of this manuscript is available on https://github.com/pmckeigue/covid-scotland_public

Please structure the abstract using the PLOS Medicine headings (Background, Methods and Findings, Conclusions - "Methods and Findings" should be a single subsection heading).

Done

In the last sentence of the Abstract Methods and Findings section, please include a brief note about any key limitation(s) of the study's methodology.

We have added a sentence to note that records from primary care were not available.

At this stage, we ask that you include a short, non-technical Author Summary of your research to make findings accessible to a wide audience that includes both scientists and non-scientists. The Author Summary should immediately follow the Abstract in your revised manuscript. This text is subject to editorial change and should be distinct from the scientific abstract. Please see our author guidelines for more information: https://journals.plos.org/plosmedicine/s/revising-your-manuscript#loc-author-summary

Done

Did your study have a prospective protocol or analysis plan? Please state this (either way) early in the Methods section.

We have inserted this sentence into the Methods: "The study followed a pre-specified protocol. Modifications after study started were that we aligned the list of conditions of interest to be consistent with those designated as moderate risk conditions by public health agencies, and extended the list of drug classes under study to include all drugs."

The authors could consider using an appropriate reporting guideline (eg, RECORD - https://www.equator-network.org/reporting-guidelines/record/ - designed to support reporting of observational studies using routinely-collected data) to enhance reporting of methods and findings in their study. If the authors choose to use this please upload the completed RECORD (or other) checklist as supporting information with the revised paper.

RECORD checklist uploaded

Note for all reviewers.

As discussed with the editor in this revised manuscript we have used an updated dataset that includes all deaths certified with COVID-19 as underlying cause, irrespective of whether a positive nucleic acid test for

SARS-CoV-2 was obtained. The data capture was extended from May 15th to June 8th.

This update has changed all the numbers in the manuscript, but none of the conclusions have changed. Our reproducible research pipeline allows us to regenerate the entire manuscript from this updated dataset with a single keystroke.

Using this update deals with several points raised by reviewers, including the inclusion of deaths among those who did not test positive, and the incomplete recording of ethnicity.

Reviewer #1:

> *Full case ascertainment of severe disease arguably limited by LTC deaths that do not reach hospital not accounted for, and may bias the rate ratios described.*

As noted above, this updated dataset includes deaths certified with COVID-19 as underlying cause among people without a positive test for SARS-CoV-2. These are tabulated separately in Supplementary Table 1.

> *Challenges are the sheer number of univariate comparisons reported, and the limited corrections for multiple comparisons in place.*

By reporting exact p–values, we allow readers to make their own assessment of the strength of evidence for association allowing for the size of the prior hypothesis space (number of independent tests). This practice is endorsed by Reviewer #2.

> *Table 5 and 6 could be placed in the supplementary appendix, unless the purpose is to emphasize the non-specificity of the associations.*

We have moved these tables to the supplementary appendix

> *The presence of less robust associations in common conditions such as ischemic heart disease, and more robust associations in rare diseases like chronic kidney disease, is notable. This is mentioned in the discussion, but should be understood better, given the conclusions.*

We have added a sentence to note the relevance of non-ischaemic heart disease and kidney disease to the involvement of these systems in severe COVID-19.

> *A great deal of the Results should be moved to the Methods, such as the multivariable model construction, and the prescription association data.*

We have moved the material describing the stepwise regression procedure to the Methods section

> *Removing neoplasms from the prespecified list because of inability to discern who has already been shielded belies the fact that shielding is i) not 100% applied in populations; ii) not practiced in other regions, making the association between neoplasm and outcome an important one to consider for policy in other regions.*

We have rewritten this sentence to clarify that neoplasms are not on the designated list of conditions that the National Health Service uses to classify "people at moderate risk", though primary neoplasms of blood or bone marrow and cancer treatments affecting the immune system are on the list used to classify "clinically extremely vulnerable" individuals who are advised to shield themselves. We have added a sentence to the Discussion to state that we are unable to investigate adequately the risk associated with neoplasms in this study, but that we hope to investigate it in a further study that will include linkage to records of shielding advice.

> *Abbreviation 'scrip' in Table 4 not universal, would use 'prescription'*

We have made this substitution.

> *Very hesitant, given the sample size, the inaccurate labelling problem, the exclusive examination of one ethnic group, and the limited generalizability, to include any reporting or conclusions on*

the ethnicity-based risk factors. Would suggest excluding altogether, and reporting on the need for more robust ethnicity-based data collection strategies

This updated dataset has more complete recording of self assigned ethnicity as the ascertained was widened to include more SMR datasets and longer lookback thus yielding data on 85% of cases and 74% of controls. Therefore we have dropped the sensitivity analysis based on name classification which lacked accuracy as noted by the referee. We now describe the numbers and rate ratios for South Asia, Black and other. We agree that the numbers are too small to make definitive conclusions. However if we do not include any data on ethnicity readers will query this given the amount of concern and publicity there has been about large ethnic differences in risk in the rest of the UK. Therefore we have made a clear statement in discussion that the numbers are too small to provide definitive statements on risk ratios.

Reviewer #2:

Page 3 I understand (probably from Sheila Bird) that death registration in Scotland is much more timely than south of the border but is there any important bias due to delays? I might just give a little bit more detail here as death registration practices are not the same all over the world.

We have edited the Methods section to give the cutoff date for ascertainment of death registrations (rather than deaths) and to note that 94% of deaths among cases were registered within 5 days.

Page 3 It took me a couple of attempts to see what the sentence starting 'By restricting' meant. How about 'Using this definition means we include all severe cases even if they die without entering critical care.' That might be too abbreviated so feel free to re–write.

We have rewritten this sentence for clarity as suggested

Page 3 In my idiolect embarkation means boarding a ship. Does this mean emigration? Whatever it means did the authors really start with 2755 strata of size 10 + 1 and end up with them all of 7 + 1 as this seems to imply. I think I have misunderstood here. I also note that (Table 1) 19670 2755 = 7.14 not 7.

We have rewritten the section on selection of matched controls. In this updated dataset the exclusion of those no longer in Scotland was done before selection of matched controls, so we are closer to the original design target of ten controls per case

Pages 4 and 5 I think it would be good to cite both gam and survival properly. It is the only credit package authors get. (Disclaimer: I am a package authors although not of these.)

We have added the citation given by the authors of the `survival` package. For the `gam` package, the author does not give a specific citation.

Page 5 If the incidence and mortality figures are a core part of the authors' thesis then I would suggest making some comparison between this and the corresponding figures for all–cause mortality. I sense though that this is perhaps just background. Is there any reason to give overall figures and then just men?

Others have compared COVID-19 mortality with all-cause mortality: a full exploration of this important question is beyond the scope of this paper.

Our figure shows mortality and incidence for men and women separately – it's possible that the lines for women were not visible to the reviewer.

Page 6 If the authors are going, correctly, to do a sensitivity analysis for different ways of identifying ethnicity then should it not be flagged in the methods? > Page 6 Despite what it says here Table 1 does show the figures for Black as well as White and South Asian and the risk ratio for Black seems indistinguishable from that for South Asian.

We have deleted the sensitivity analyses regarding using name-based ethnicity classification as we now have more complete recording of self assigned ethnicity by doing longer and wider lookback in SMR records. We now report for White, South Asian, Black and other categories.

> *Page 6 The authors state here that Table 3 shows listed conditions broken down by age (so that we see that immune conditions were not more common in 75+). I do not see such a breakdown there.*

We have corrected this table reference

> *Page 7 The phrase 'most significant association was with diagnoses in ICD chapter 2' seems to equate the smallness of the p–value with importance since several of the other risk ratios in Table 5 are of comparable size or even greater than that for neoplasms.*

We have rewritten this.

> *Page 7 Viewing this from the perspective of a future researcher who finds something apparently different in their data I wonder whether the criteria for Table S5 are too restrictive. I can understand excluding rare conditions (the current limit is 50) but if you restrict by p–value people who get a similar risk ratio to the present authors will not be aware of the similarity if the current authors' p–value was > 0.001.*

We have filtered this table by p-value to keep the length of the table within reasonable limits. We plan to make more detailed summary statistics available elsewhere.

> *Page 8 One bit does not seem very much information to me and I find it hard to see how it would work in practice.*

We have added a more detailed explanation of this under "Relevance to policy" in the Discussion – the total information for discrimination is the sum of the information from the logistic regression on age and sex and the information from the conditional logistic regression on risk factors, conditioned on age and sex.

> *Page 8 While it is welcome to see the authors challenge the notion that everybody whose family origins are in South Asia is the same I wonder how much sense this is going to make to people outside the UK or South Asia. International readers may not know how and why people from the disparate communities came to the UK nor why this issue seems so salient in the UK discourse about COVID–19.*

Given the comments of Reviewer #1 we have shortened this part of the discussion making clearer the background concern from other studies in the UK but keeping our inference about the data in Scotland brief and noting the wide confidence intervals.

> *Page 14 I assume the black dots are hidden behind the black curve? Strictly speaking multivariable is meant throughout I think (Hidalgo and Goodman, 2013). Multivariate means multiple variables on the left hand side which is not, as far as I can see, true of the models used. I know this is very picky.*

We have substituted the term multivariable for multivariate.

> *The authors comment on page 8 that they have shown that the risk of a severe outcome increases with age. As far as the death component is concerned that was true of course before this particular virus appeared. According to the English Life Tables ELT17 the value of mx for a male my age was 0.025 and for a male ten years older than me 0.075 which is the same sort of increase with age as the authors are reporting for their severe outcome. What we do not know is what the excess risk is over and above the background risk and how that varies with age.*

We have added an explanation in the Discussion of how rate ratios can be interpreted as increases in "Covid age".

Reviewer #3

> *It would be informative if the authors could break down the severe cases into those that were due to death within 28 days without admission to critical care units, those that were admitted to critical care units and survived, those admitted to critical care but perished. It would be interesting to analyze whether what proportion of severe cases among care home residents were death within 28 days without admission to critical care units. Providing the severe cases breakdown and a more in-depth discussion will further strengthen the conclusions.*

We have added a supplementary table S1 to provide this breakdown of severe cases, and described what it shows in the first paragraph of Results. As Reviewer #3 suggested, this shows clearly that almost all fatal cases in care home residents did not enter critical care.

> *Since the authors concluded that many preexisting conditions were associated with severe COVID-19, may consider adding ANY comorbidity in Table 3.*

The "any comorbidity" variable is implicit in the variable "any prescription or diagnosis". Other than diabetes which was ascertained from a population register, all other comorbidities are based on dispensed prescriptions or hospital diagnoses.
